# Advances in MicroRNA-Mediated Reprogramming Technology

**DOI:** 10.1155/2012/823709

**Published:** 2012-03-28

**Authors:** Chih-Hao Kuo, Shao-Yao Ying

**Affiliations:** Department of Cell and Neurobiology, Keck School of Medicine, University of Southern California, BMT-403, Los Angeles, CA 90033, USA

## Abstract

The use of somatic cells to generate induced-pluripotent stem cells (iPSCs), which have gene characteristic resembling those of human embryonic stem cells (hESCs), has opened up a new avenue to produce patient-specific stem cells for regenerative medicine. MicroRNAs (miRNAs) have gained much attention over the past few years due to their pivotal role in many biological activites, including metabolism, host immunity, and cancer. Soon after the discovery of embryonic-stem-cell- (ESC-) specific miRNAs, researchers began to investigate their functions in embryonic development and differentiation, as well as their potential roles in somatic cell reprogramming (SCR). Several approaches for ESC-specific miRNA-mediated reprogramming have been developed using cancer and somatic cells to generate ESC-like cells with similarity to iPSCs and/or hESCs. However, the use of virus-integration to introduce reprogramming factors limits future clinical applications. This paper discusses the possible underlying mechanism for miRNA-mediated somatic cell reprogramming and the approaches used by different groups to induce iPSCs with miRNAs.

## 1. Introduction

In the 1980s, the first mouse embryonic stem cell line, which displayed both the capability for unlimited proliferation and the pluripotency and capacity to differentiate into three germ layers, was established, and since then, embryonic stem cells have shown great promise for advancing the fields of drug discovery, disease modeling, and regenerative medicine. It was believed that cells differentiated in a unidirectional manner until the invention of somatic cell nuclear transfer (SCNT) [1]. This technique replaces the nuclei of oocytes with those of somatic cells, resulting in the reversal of the fused cells from a differentiated to pluripotent status. Subsequently, many species of mammalian cells have been successfully cloned, including the famous cloned sheep Dolly [2]. However, this approach raises problems, such as the ethical concerns regarding the use of embryos and the technical challenges and low success rate associated with this method.

In 2006, Takahashi and Yamanaka successfully generated induced-pluripotent stem cells (iPSCs) with a novel approach using the viral integration of the four transcription factors, Oct4, Sox2, Kl4, and c-Myc [3]. This revolutionary approach has demonstrated the feasibility of using somatic cells to generate iPSCs. Patient-specific iPSCs can be derived from cells of the same patient, thereby avoiding the immune rejection that occurs with SCNT or human embryonic stem cells (hESCs). More importantly, use of somatic cells as the starting material circumvents the ethical issues associated with the use of human embryos. Subsequently, many iPSCs have been generated from various types of somatic cells, including keratinocytes, neuronal stem cells, and fibroblasts, among others [3–6]. These iPSCs display gene expression patterns, cell morphology, and the capacity of forming teratoma *in vivo* similar to those of hESCs [3, 6]. Nevertheless, the use of retroviral delivery hinders its application in clinical therapy because integration of viral genes into the iPSC genome may cause instability that leads to undesired mutations [7]. In addition, studies have shown that the ectopic expression of c-Myc, one of the original four transcription factors, correlates with increased tumorigenicity and further raises questions about the therapeutic potential of iPSC generated in this manner [8]. Although successful reprogramming has been reported using only 2 Yamanaka factors in the absence of c-Myc, this approach results in greatly decreased efficiency [9]. In addition, synthetic modified mRNAs have been used to replace viral delivery for elevating expression of the four Yamanaka factors, and while these studies demonstrated the efficient reprogramming of cells in the absence of retrovirus, ectopic c-Myc was still present [10, 11].

In 2004, Suh and colleagues identified a set of embryonic stem cell-specific microRNAs (miRNAs), [12] which have subsequently been found to contribute to embryo development [13–15]. In fact, deficiency in theses miRNAs can cause detrimental defects in cell proliferation and differentiation [16, 17]. Among the highly expressed ESC-specific miRNAs, miR-302/367 is highly expressed in early embryonic development and then rapidly declines after differentiation [12, 13]. This information prompted several laboratories to investigate the role of miR-302/367 in reprogramming [18–20]. Numerous miRNA-mediated iPSC lines have been subsequently developed with either miR-302/367 or a combination of miR-302 and other miRNAs from mouse fibroblast, human dermal fibroblast, and human skin cancer cells [21, 22]. As this approach avoids the use of oncogene c-Myc, the possibility exists that miRNA-reprogrammed iPSCs would be more suitable for human use.

## 2. Proposed Mechanism of miR-302/367-Mediated Reprogramming

Currently, the mechanism by which somatic cells generate iPSCs remains unclear. Maternal materials rather than downstream transcription factors regulate the maintenance and renewal of fertilized oocytes [23]. A large portion of RNAs in mouse oocytes are transcribed from the maternal genome, and maternal miRNAs present in the oocyte rapidly decline during the oocyte-zygote transition. Therefore, it is reasonable to assume that these maternal miRNAs inhibit the developmental signal for maintaining pluripotency in the early embryonic stage through an RNAi effect. This, in combination with the observation of high miR-302/367 expression in the early embryo followed by a rapid decrease upon differentiation, strongly suggests that miR-302/367 serves as upstream pluripotency regulator to modulate the expression of Oct4, Sox2, Nanog, and other embryonic transcription factors. Given that miRNA can target several to hundreds of genes, the inhibition of multiple factors and pathways likely initiates miR-302/367 reprogramming.

miR-302/367 targets multiple epigenetic factors, leading to global demethylation. Global DNA demethylation occurs at the promoter binding site of several ESC-specific transcription factors during the 1–8 cell stages of early zygotes, resulting in preservation of imprinting. The same mechanism very likely happens during somatic cell reprogramming (SCR). MiR-302 silences lysine-specific histone demethylases 1 and 2 (AOF1 and AOF2) and methyl-CpG-binding proteins 1 and 2 (MECP1-p66 and MECP2). DNA methyltransferase 1 (DNMT1), an essential regulator in DNA methylation, is then silenced in response to the downregulation of AOF2, leading to genomewide demethylation and consequently coactivation of pluripotency-promoting genes [20, 24]. Indeed, silencing AOF2 enhanced global demethylation during reprogramming of human hair follicle cells by miR-302s [20].

miR-302/367 also directly targets NR2F2, a member of the nuclear orphan receptor family of transcriptional factors and a negative regulator of Oct4 [25]. In hESCs, NR2F2 expression begins with differentiation and conversely correlates with the expression of Oct4 and miR-302/367. Studies have also shown that Oct4, Nanog, and Sox2 bind to the promoter regions of miR-302/367 and increase its expression level [26]. Taken together, miR-302/367 expression induces global demethylation and suppresses NR2F2, two events that indirectly activate Oct4 expression, which in turn elevates miR-302/367 levels. This reciprocal cycle increases cellular levels of miR-302/367 and Oct4, which leads to the co-activation of other transcription regulators, such as Sox2 and Nanog. Studies from Lin and colleagues showed that overexpression of mir-302/367 (approximately 1.1- to 1.3-fold as compared with normal hESCs) leads to global demethylation and coexpression of Oct4, Sox-2, and Nanog in human iPSCs [20, 27]. A similar study using RUES2 cells confirmed that transfection with miR-302 elevates Oct4 and Nanog expression [14].

Additional targets may include the transforming growth factor beta receptor II (TGFBR2) and ras homolog gene family member C (RHOC) genes. A recent study has reported that a combination of miR-302b and miR-372 downregulates TGFBR2 and RHOC gene expression [19]. Furthermore, the inhibition of these two molecules correlates with increased efficiency of iPSC induction [19]. Evidence has shown that mesenchymal-to-epithelial transition (MET) occurs during the early reprogramming process in mouse fibroblasts as a result of blocking pro-epithelial-to-mesenchymal signals, such as TGF-beta [28]. The same process may also occur during reprogramming in human cells. We found that the miR-302 may also target TGFBR2 and RHOC, supporting the possibility that miR-302/367 has the same effect on MET as miR-302b/327. However, confirmation of this hypothesis requires further study.

miRNA-reprogrammed iPSCs display a decrease in tumorigenecity as compared with the iPSCs generated by conventional approaches [27], which is likely due to multiple factors. For example, miRNA-mediated somatic cell reprogramming does not require enhanced expression of c-Myc; therefore, no oncogene is involved in the process. Also, miR-302/367 targets several cell cycle regulators. During miRNA-mediated SCR, both cyclin E-CDK and cyclin D-CDK4/6 undergo downregulation. Consequently, this attenuates the G1 to S phase transition, resulting in decreased iPSCs tumorigenicity. In addition, two tumor suppressor genes, p16Ink4a and p14/19Arf, undergo upregulation through the silencing of BMI1 (B lymphoma Mo-MLV insertion region 1 homolog). Taken together, the cell cycle of microRNA-reprogrammed iPSCs is highly regulated and resembles the early mammalian zygote (20 to 24 hours) [27].

## 3. Different MicroRNA Approaches for SCR

In order to study the role of miRNAs in reprogramming as well as in other physiological events, it is crucial to develop an artificial expression vector that can both generate functional mature miRNAs and maintain their expression *in vitro* and *in vivo*. Previously, several vectors have been established that use RNA polymerase III transcription activity to generate stable miRNA expression [29–32]. These vectors, however, have several drawbacks. First, the ubiquity of pol III makes it is difficult to target a specific population of cell types. Second, transcription by RNA pol III requires U6 and H1 promoters. Pol III activity could potentially generate the accumulation of large RNA transcripts (>25 bps), thereby producing interferon cytotoxicity [33, 34]. A recombinant gene expression system, mediated by RNA polymerase II and based on the mechanism of miRNA biogenesis, has also been developed [35–37]. The precise regulation of RNA splicing and nonsense-mediated decay of pol-II-directed RNA biogenesis ensures the degradation of excessive RNA accumulation and alleviates potential toxicity caused by high levels of long double-stranded RNAs [38, 39]. miRNAs transcripts are frequently located in an intron in proximity to the protein coding region; thus, this expression system uses an artificial intron (SpRNAi) placed between two exons that, together, encode a reporter, such as green or red fluorescent protein. Because the intronic miRNA of interest disrupts the functional structure of the reporter gene, expression will not occur without proper RNA splicing. Detection of the fluorescent signal provides a means to monitor the production of this intronic miRNA. The artificial intron (SpRNAi) consists of the following components: a 5′-splice site, a branch-point domain, a polypyrimidine tract, and a 3′-splice site, and the pre-miRNA insert is placed between the 5′-splice site and branch-point domain. This system has been shown to induce the RNAi effect in LNCaP, HeLa, and HCN-A94-2 cells as well as in mice [40, 41].

To date, viral transfection serves as the primary method to introduce reprogramming factors, either Yamanaka factors or miRNAs, into cells due to its high efficiency of delivery. However, as mentioned previously, this may result in the integration of exogenous genes into the host genome and as such is not ideal for clinical trials. Many studies have been conducted to find alternatives with higher transduction efficiency. A recent study examined mature miRNAs rather than vector-based delivery as a potential approach for SCR [18]. The use of mature miRNA bypasses the DNA-based plasmid and thus avoids any possible insertion of genes into genome of targeted cells. Further, higher efficiency (0.1%) has been achieved as compared with the retroviral delivery of the Yamanaka factors (0.01~0.04%). Although this method seems promising, several problems remain. Depending on the cell type and experimental conditions, SRC requires sufficient amounts of cellular mature miRNAs over the course of days to weeks. Thus, repeated transfections may be necessary to maintain the appropriate levels over time. The need for ample amounts of synthetic nucleotides may greatly increase the cost for the large-scale production of iPSCs in a clinical setting. Moreover, instead of the miR-302 family alone, it has been argued that successful reprogramming by mature miRNAs always requires the combination of miR-302 s, miR-200c, and miR-369, while several miRNA-mediated iPSC lines have been generated with only miR-302 or miR-302/367 [20, 22].

## 4. Conclusions

Based on evidence of the successful establishment of iPSC lines using a miRNA-mediated strategy, it seems that ESC-specific miRNA, especially the miRNA-302/367 family, can induce reprogramming events similar to those of Yamanaka factors. The use of a miRNA expression vector, such as the intronic miRNA expression system, provides a simple and safe way to generate iPSCs due to the fact that no oncogene is required for successful reprogramming and, in the case of miR-302/367, only a single transcript is transfected rather than the simultaneous transfection of multiple genes, whose expression would be difficult to consistently maintain in iPSCs. In general, delivery of premade miRNAs provides a fast and direct way to recapitulate miRNA-mediated RNAi as compared to a vector-based approach. While its practicality for large-scale production of patient-specific iPSCs remains to be determined, the potential underlying mechanism of miRNA-mediated reprogramming suggests that it may represent an improved means to generate patient-specific iPSCs, with better quality and safety for regenerative medicine and transplantation therapy.
